# Convergent evolution revealed by paraphyly and polyphyly of many taxa of oribatid mites: A molecular approach

**DOI:** 10.1007/s10493-024-00960-1

**Published:** 2024-09-08

**Authors:** Peter Cordes, Xue Pan, Maka Murvanidze, Anna Seniczak, Stefan Scheu, Ina Schaefer, Mark Maraun, Bastian Heimburger

**Affiliations:** 1 J.F. Blumenbach Institute of Zoology and Anthropology, University of Göttingen, 37073 Göttingen, Germany; 2 Tbilisi State University, 0179 Tbilisi, Georgia; 3 Centre of Biodiversity and Sustainable Land Use, University of Göttingen, 37077 Göttingen, Germany; 4 Agricultural Sciences and Biotechnology, Inland Norway University of Applied Sciences, Elverum, Norway; 5 Senckenberg Biodiversity Climate Research Center, Frankfurt Main, Germany; 6 LOEWE Center for Translational Biodiversity Genomics (LOEWE-TBG), Frankfurt Main, Germany

**Keywords:** Convergent evolution, Arachnida, Phylogeny, Oribatida, Endeostigmata, 18S rDNA

## Abstract

A reliable phylogeny is crucial for understanding the evolution and radiation of animal taxa. Phylogenies based on morphological data may be misleading due to frequent convergent evolution of traits—a problem from which molecular phylogenies suffer less. This may be particularly relevant in oribatid mites, an ancient soil animal taxon with more than 11,000 species, where the classification of species into high-ranking taxa such as superfamilies is equivocal. Here, we present a molecular phylogeny of 317 oribatid, 4 astigmatid and 17 endeostigmatid mite species/taxa based on 18S rDNA sequences. We aimed at testing the validity of the 41 superfamilies of oribatid mites recognized by Norton and Behan-Pelletier (in Krantz and Walter, A manual of Acarology, 3rd ed., Texas Technical University Press, Lubbock, 2009). The results indicate that 17 of the 41 oribatid mite superfamilies are monophyletic but that 18 superfamilies are paraphyletic or polyphyletic (5 superfamilies were only included with one species and Microzetoidea were not included). Our findings point to the importance of convergent evolution in polyphyletic oribatid mite taxa. Convergent evolution and the old age of mites likely resulted in a mosaic-like distribution of morphological characters impeding phylogenetic reconstructions based on morphology, calling for molecular approaches to improve oribatid mite systematics.

**Supplementary Information:**

The online version contains supplementary material available at 10.1007/s10493-024-00960-1.

## Introduction

To understand the evolution of soil animal biodiversity, a solid phylogeny is required (Greisen et al. 2019; Guerra et al. 2020). Considering the frequency of convergent evolution, molecular methods are a suitable tool (McGhee 2011; Mushtaq et al. 2023). Particularly in ancient groups such as mites (Acari) the use of molecular approaches offers the best perspective for resolving their natural history (Schaefer et al. 2010; Arribas et al. 2020).

Acari (Chelicerata) comprise more than 50,000 described species (Zhang 2011), while the true number of species may exceed one million (Alberti 2006). Despite more than a century of Acari taxonomy (Berlese 1897; Oudemans 1923), grouping of taxa into monophyletic lineages remains challenging (Dunlop et al. 2014; Lozano-Fernandez et al. 2019; Schäffer et al. 2020; Pachl et al. 2021). Monophyly of the two main mite taxa, i.e. Acariformes and Parasitiformes, is widely accepted (Krantz and Walter 2009) and confirmed by molecular data (Arribas et al. 2020; Li and Xue 2019). However, the monophyly of individual higher taxa within Acariformes, such as oribatid mites (Oribatida) and endeostigmatid mites (Endeostigmata), is still debated (Dabert et al. 2010).

Oribatid mites are globally distributed and comprise more than 11,000 described species (Subías 2004, 2022; Schatz 2004), but their actual diversity certainly is much higher (Travé et al. 1996; Schatz 2002). Most species (~ 7000) belong to the derived Brachypylina (Schatz et al. 2011; Subías 2004, 2022), with the more basal oribatid mites being grouped into Palaeosomatides, Enarthronotides, Parhyposomatides, Mixonomatides and Desmonomatides (= sensu Nothrina) (Norton and Behan-Pelletier 2009; Schatz et al. 2011; Subías 2004, 2022). Brachypylina are characterized by having adults with a cap-like notogaster that is isolated from a one-piece venter and prodorsum (Weigmann 2006). Major morphological characters that distinguish oribatid mite lineages include (i) dermal glands, (ii) the form of the chelicerae, and (iii) juvenile morphology (Weigmann 2006; Norton and Behan-Pelletier 2009; Weigmann and Ermilov 2016). However, many characters are not lineage-specific and their use for assigning species to higher-ranked taxa such as superfamilies remains ambiguous (Norton and Behan-Pelletier 2009). In his list of oribatid mite species of the world, Subías (2022) distinguished 163 families and 1013 genera, and classified them into 51 superfamilies. By contrast, Schatz et al. (2011) distinguished 174 families and 1259 genera, and classified 42 lineages as superfamilies. Similarly, Norton and Behan-Pelletier (2009) recognized 41 lineages as superfamilies.

Endeostigmatid mites are a paraphyletic group of acariform mites with many plesiomorphic characters, and some taxa likely being part of Oribatida (Dabert et al. 2010; Pepato and Klimov 2015). Typically, they are small and inhabit extreme habitats such as deserts, sandy soils or deep soil layers (Walter 2009).

Our principal aim was to test the monophyly of the 41 oribatid mite superfamilies recognized by Norton and Behan-Pelletier (2009) using 18S rDNA sequences. This gene has been used before to reconstruct the phylogeny of chelicerates (Turbeville et al. 1991) and oribatid mites (Maraun et al. 2009; Schaefer et al. 2010; Schäffer et al. 2020; Pachl et al. 2021), and sequence data present in GenBank represent most of the targeted 41 oribatid mite lineages. We included representatives of Palaeosomatides, Enarthronotides, Parhyposomatides, Mixonomatides and Desmonomatides and covered all 24 brachypyline superfamilies proposed by Norton and Behan-Pelletier (2009). Furthermore, we investigated whether the five superfamilies of Endeostigmata, i.e., Alicorhagioidea, Alycoidea, Nematalycoidea, Oehserchestoidea and Terpnacaroidea (Walter et al. 2011) are monophyletic. We did not include Eriophyoidea although they have recently been proposed to be closely related to Nematalycidae (Endeostigmata) (Bolton et al. 2023) since it was not the aim of our study to resolve phylogenetic relationships of Endeostigmata.

## Material and methods

Taxon sampling included 317 oribatid mite species, representing Palaeosomatides, Enarthronotides, Parhyposomatides, Mixonomatides, Desmonomatides and Brachypylina. Furthermore, we included 17 taxa of Endeostigmata covering Alycoidea, Nematalycoidea, Oehserchestoidea, Terpnacaroidea and Alicorhagioidea (Walter et al. 2011) and four Astigmata taxa including *Neottialges vitzthumi*, *Austroglycophagus geniculatus*, *Nanacarus* spec., and *Histiostoma inquilinum*. *Opilioacarus texanus* (Opilioacarida) was used as outgroup. Sequence data was obtained from NCBI GenBank (www.ncbi.nlm.nih.gov). If more than one sequence was available for any mite taxon, we aligned all available sequences and selected the longest sequence with the fewest ambiguous positions (see supplementary file 1). In addition to taxa that were available from GenBank (see supplementary Table 1), the oribatid mite species *Diapterobates humeralis*, *Eueremaeus silvestris*, *Euzetes globulus*, *Licneremaeus licnophorus*, *Melanozetes mollicomus*, *Minunthozetes semirufus*, *Mycobates sarekensis*, *Oribatella quadricornuta*, *Svalbardia bicuspidata*, *Punctoribates sellnicki* and *Podopterotegaeus bisetus* were sequenced for this study. Template DNA was extracted from five pooled individuals using DNeasy Blood and Tissue Kit (Qiagen, Hilden, Germany) following the manufacturer’s protocol, but using 30 µl elution buffer. Animals were crushed using a pestle, and lysed for 4 h at 56°C. The 18S rDNA region was amplified in three segments with the following primer pairs: 1st segment 18S f 5′- ACCTGGTTGATCCTGCCAG-3′, 18S 614r 5′-TCCAACTACGAGCTTTTTAACC-3′; 2nd segment 18S 554f 5′-AAGTCTGGTGCCAGCAGCCGC-3′, 18S 1282r 5′-TCACTCCACCAACTAAGAACGGC-3′; 3rd segment 18S 1150f 5′-ATTGACGGAAGGGCACCACCAG-3′, 18SR 5′- TAATGATCCTTCCGCAGGTTTCAC-3′ (Domes et al. 2007a, b). The PCR mastermix included 12.5 µl SuperHot Taq Mastermix (Genaxxon, Ulm, Germany), 1.5 µl MgCl_2_ (25 mM), 1 µl of each primer (10 pM), 1 µl BSA (~ 3%) and 4 µl of template DNA. The PCR protocol contained the following steps: (1) Initial activation at 95 °C for 15 min; (2) 35 amplification cycles and denaturation at 95 °C for 15 s; (3) annealing at 57 °C (1st and 3rd primer pair), and 59 °C (2nd primer pair) for 60 s; (4) elongation at 72 °C for 60 s; (5) final elongation at 72 °C for 10 min. Successfully amplified PCR products were purified using the PCR-DNA Purification Mini Spin Column Kit (Genaxxon, Ulm, Germany) and sequenced at Microsynth Seqlab after Sanger (Göttingen, Germany).

Sequences were aligned using MAFFT v7.490 (Katoh et al. 2002; Katoh and Standley 2013) implemented in Geneious Prime 2024.0.5 (www.geneious.com) using default settings. The total alignment length was 2628 base pairs (bp). We removed gap positions using the gappyout option in trimAl v1.4.rev15 (Capella-Gutierrez et al. 2009), which automatically removes gaps by calculating and sorting gap scores for each column and plotting potential gap score thresholds against the percentage of alignment not reaching these thresholds (Capella-Gutierrez et al. 2009). For the final alignment with a length of 1838 bp see supplementary file 2. A Maximum-Likelihood tree was calculated with IQ-TREE v2.3.2 (Nguyen et al. 2015) using GTR + G + I as substitution model and 10 independent runs (‘--runs 10’). We performed 1000 replicates each for both ultrafast bootstraps (ufBS) (‘-bb 1000’) and SH-aLRT tests (‘-alrt 1000’). Nodes were classified as “robust” if recovered support values for ufBS and SH-aLRT test were ≥ 95% and ≥ 80%, respectively.

The tree was visualized using the R packages ggplot2 (Wickham 2016) and ggtree (Yu et al. 2017) in the R software v4.1.2 with the R studio interface (The R Foundation for Statistical Computing 2021). We tested monophyly for 35 of the 41 oribatid mite lineages. Microzetoidea were not included in our analyses and Eulohmannioidea, Perlohmannioidea, Nehypochthonioidea, Eremaeozetoidea and Polypterozetoidea were each represented by only a single species/sequence.

We conducted a tree topology test, specifically an Approximately Unbiased (AU) test (Shimodaira 2002), utilizing both our reconstructed tree and a hypothetical tree (Appendix; Fig. S1). This hypothetical tree illustrates the relationships among the 41 oribatid mite superfamilies as described by Norton and Behan-Pelletier (2009). The hypothetical tree was generated using IQ-TREE with the same alignment, substitution model and parameters as used previously, but with the constrained tree search option ('-g'). The AU test was performed using IQ-TREE with zero search iterations and 10,000 RELL (Kishino et al. 1990) replicates ('-n 0 -zb 10,000 -au').

## Results

**Fig. 1:**
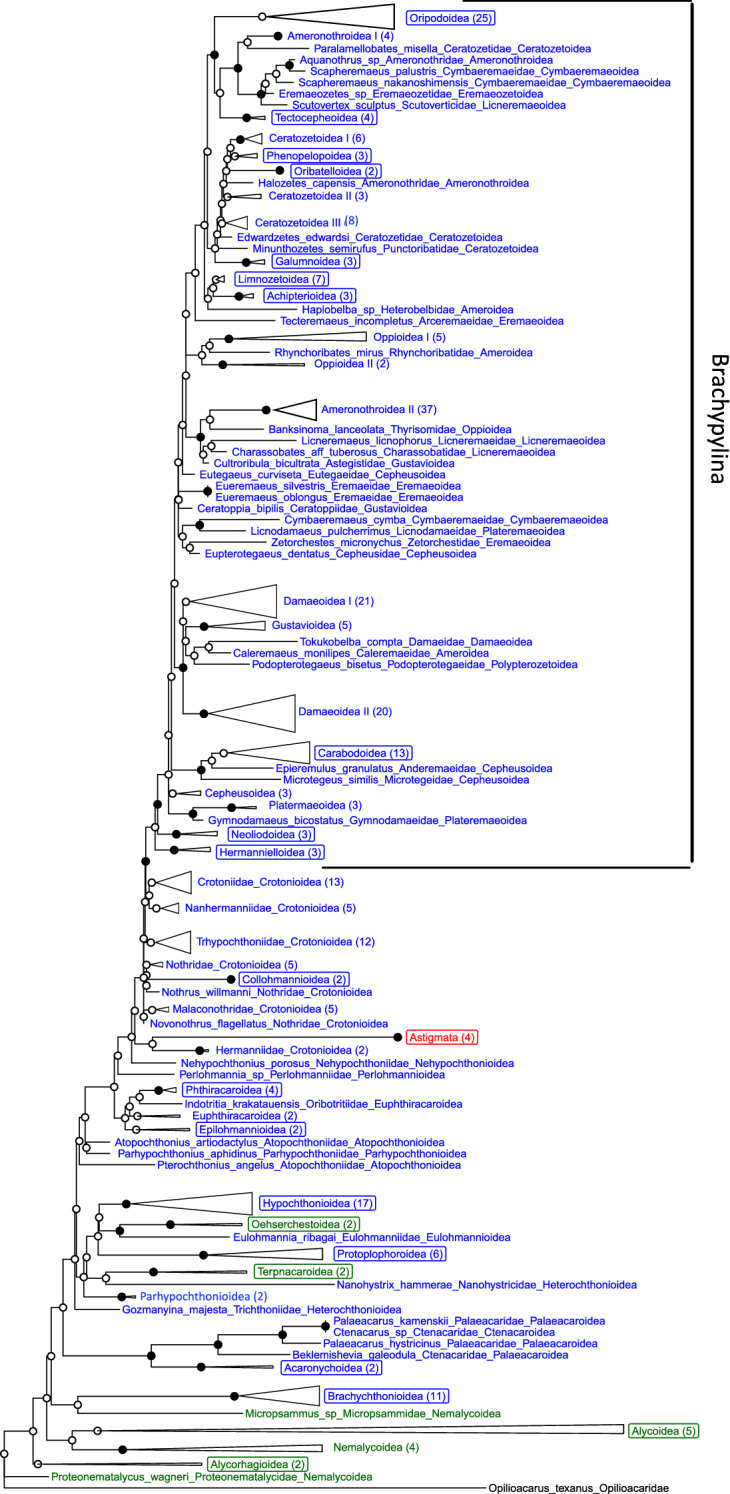
Maximum Likelihood phylogeny of oribatid and endeostigmatid mite taxa based on 18S rDNA sequences. Astigmata taxa included *Neottialges vitzthumi*, *Austroglycophagus geniculatus*, *Nanacarus* spec., and *Histiostoma inquilinum*. *Opilioacarus texanus* (Opilioacarida) was used as outgroup. Overall, 319 mite taxa were included in this study (see Appendix, Fig. S2) which were collapsed to 119 taxa. The monophyletic Astigmata, 17 monophyletic oribatid and four monophyletic endeostigmatid mite lineages (‘superfamilies’ according to Norton and Behan-Pelletier 2009) are framed in red, blue and green, respectively. The scale indicates genetic distance, i.e., nucleotide substitutions per site, numbers in brackets indicate the number of included taxa in a collapsed clade. Oribatid mites are marked in blue, Astigmata in red and Endeostigmata in green. Names of monophyletic superfamilies are in a box. Black circles indicate nodes that were supported by both, SH-aLRT (≥ 80) and ultrafast bootstrap (≥ 95) analyses

Phylogenetic analyses indicated that 17 of 41 oribatid mite superfamilies and four of the five Endeostigmata lineages were monophyletic. Of the 17 non-brachypyline oribatid mite superfamilies seven are indicated to be monophyletic, as well as 10 of the 24 brachypyline superfamilies (see Fig. 1 for the collapsed phylogenetic tree and Appendix, Fig. S3, for the uncollapsed phylogenetic tree). The AU test significantly rejected the hypothetical tree (p-AU; P < 0.001), which is also reflected in ufBS and SH-aLRT support values for both trees (Appendix; Figs. S2 and S3).

### Non-brachypyline lineages

The seven monophyletic non-brachypyline superfamilies included (1) Acaronychoidea (100/100; ufBS/SH-aLRT), (2) Brachychthonioidea (100/100), (3) Collohmannioidea (100/100), (4) Hypochthonioidea (96.5/98), (5) Phthiracaroidea (97.3/100) and (6) Protoplophoroidea (100/100) (Fig. 1). For (7) Epilohmannioidea (89.2/92) the support was less strong.

As indicated by our phylogeny, the following seven non-brachypyline superfamilies are not monophyletic. Members cluster together with species from other superfamilies, usually with high support (Fig. 1). (1) Atopochthonioidea were indicated to be diphyletic due to *Atopochthonius artiodactylus* and *Pterochthonius angelus* being separated. (2) Ctenacaroidea and (3) Palaeacaroidea are paraphyletic due to *Beklemishevia galeodula* (Ctenacaroidea) forming the sister taxon (98.3/100) to a clade (99.9/100) comprising *Palaeacarus hystricinus* (Palaeacaroidea) + *Ctenacarus* spec. (Ctenacaroidea) + *Palaeacarus kamenskii* (Palaeacaroidea). (4) Crotonioidea are paraphyletic, since species belonging to Collohmannioidea grouped together within Crotonioidea, albeit with very weak support (0/78), and since some taxa of Crotonioidea were sister taxa to Brachypylina. (5) Euphthiracaroidea are paraphyletic due to *Indotritia krakatauensis* (Oribotritiidae) being separated from *Synichotritia caroli* (Synichotritiidae) and *Rhysotritia duplicata* (Euphthiracaridae) that formed a well-supported clade (89/100). (6) Heterochthonioidea are diphyletic due to *Nanohystrix hammerae* (Nanohystricidae) and *Gozmanyina majestus* (Trichthoniidae) being separated. (7) Parhypochthonioidea (including Gehypochthoniidae and *Parhypochthonius aphidinus*) were also diphyletic. Nehypochthonioidea, Perlohmannioidea and Eulohmannioidea were only represented by a single species.

### Brachypylina

As indicated by our phylogeny, Brachypylina are monophyletic with high nodal support values (95.3/99) (Fig. 1). Within Brachypylina, monophyly of nine of the 24 superfamilies was strongly supported, i.e. (1) Achipterioidea (100/100), (2) Carabodoidea (99.3/100), (3) Galumnoidea (99.7/100), (4) Hermannielloidea (99.1/100), (5) Neoliodoidea (99.4/100), (6) Oribatelloidea (100/100), (7) Oripodoidea (100/97), (8) Phenopelopoidea (92.5/100) and (9) Tectocepheoidea (97.3/100), whereas the support for Limnozetoidea was weaker (78.9/88).

On the other hand, our phylogeny indicated that eleven of the 24 brachypyline superfamilies are not monophyletic because members clustered with species from other superfamilies with high support (Fig. 1). (1) Ameroidea were polyphyletic with *Caleremaeus monilipes* (Ameroidea) in Damaeoidea making the latter paraphyletic, with *Rhynchoribates mirus* (Ameroidea) clustering in the paraphyletic Oppioidea and Heterobelbidae (*Haplobelba* spec., Ameroidea) being the sister taxon of Tectocepheoidea (43.6/76). (2) Ameronothroidea were polyphyletic, being dispersed in the tree among four different branches with high support, i.e. Fortuyniidae + Selenoribatidae (99.9/100), Ameronothridae (98.5/100) and the single species *Aquanothrus* spec. and *Halozetes capensis*. (3) Cepheusoidea were paraphyletic since only three of the seven studied taxa of Cepheusidae clustered together (86.8/99). (4) Ceratozetoidea were paraphyletic, species of this superfamily dispersed among four different branches of the tree. (5) Cymbaeremaeoidea were paraphyletic with the two *Scapheremaeus* species *(*Cymbaeremaeidae*)* being separated from *Cymbaeremaeus cymba* (Cymbaeremaeidae). (6) Damaeoidea were paraphyletic as the branch included *Caleremaeus monilipes* (Ameroidea) (99.1/99). (7) Eremaeoidea were indicated to be polyphyletic with the families Tecteremaeidae (*Tecteremaeus incompletus*) and Zetorchestidae (*Zetorchestes micronychus*), and being separated from the Eremaeidae (100/100). (8) Gustavioidea were polyphyletic as they included one clade (86.2/99) with Xenillidae (*Xenillus discrepans* and *X. tegeocranus*), Liacaridae (*Adoristes ovatus*) and Tenuialidae (*Hafenrefferia gilvipes*) and two phylogenetically separated lineages, i.e. the ceratoppiid species *Ceratoppia bipilis* and the astegistiid species *Cultroribula bicultrata*. (9) Licneremaeoidea were polyphyletic with Charassobatidae (*Charassobates* aff. *tuberosus*) and Licneremaeidae (*L. licnophorus*) clustering together with *Cultroribula bicultrata* (Gustavioidea), however, only with moderate support (73.9/59). Furthermore, Scutoverticidae (*Scutovertex sculptus*) were separated from the other Licneremaeoidea. (10) Oppioidea were polyphyletic since *Rhynchoribates mirus* (Ameroidea) clustered inside Oppioidea, and *Banksinoma lanceolata* (Oppioidea) was associated with Ameronothroidea. (11) Plateremaeoidea were diphyletic with the licnodamaeid *Licnodamaeus pulcherrimus* being separated from a cluster of Gymnodamaeidae (99.7/100). The monophyly of two superfamilies, i.e. Polypterozetoidea and Eremaeozetoidea, could not be evaluated as they were represented by a single species, each.

### Endeostigmata

Our phylogeny indicated Endeostigmata to be paraphyletic (Fig. 1). Monophyly of two of the five Endeostigmata superfamilies was strongly supported i.e., Oehserchestoidea (99.9/100) and Terpnacaroidea (97/100), whereas the support for Alycoidea (83.2/41) and Alycorhagioidea (56.3/60) was less strong. The paraphyletic Endeostigmata included Nematalycoidea with the families Proteonematalycidae (*Proteonematalycus wagneri*) and Micropsammidae (*Micropsammus* spec.) being separated from the other Nematalycoidea.

## Discussion

Overall, the results indicate that seven of the 17 non-brachypyline and ten of the 24 Brachypylina superfamilies proposed by Norton and Behan-Pelletier (2009) are monophyletic, whereas seven non-brachypyline and eleven of the 24 Brachypylina are likely to be para- or polyphyletic. Three non-brachypyline taxa and two Brachypylina were only represented by a single species and Microzetoidea were not included.

### Monophyletic non-brachypyline lineages

*Desmonomatides.* Although Collohmannioidea formed a monophyletic clade, they grouped with Desmonomatides rather than Mixonomatides as suggested earlier (Grandjean 1969; Norton and Behan-Pelletier 2009; Norton and Sidorchuk 2014). This grouping indicates that opisthonotal glands in *Collohmannia gigantea* (Raspotnig et al. 2001, 2003), Desmonomatides and Astigmata only evolved once arguing for their close relationship as suggested earlier (Sakata and Norton 2001).

*Enarthronotides.* Supporting our results, monophyly of Brachychthonioidea, Hypochthonioidea and Protoplophoroidea has been corroborated by morphological (Norton et al. 1983; Norton 1984, 2001) and molecular data (Maraun et al. 2004; Arribas et al. 2020; Pachl et al. 2021). Monophyly of Hypochthonioidea supports earlier results that ptychoid body forms in Mesoplophoridae (Hypochthonioidea) and Protoplophoridae (Protoplophoroidea) evolved convergently (Pachl et al. 2012). Monophyly of Protoplophoroidea supports the notion that the pharyngeal complex is a synapomorphy of this lineage (Norton and Behan-Pelletier 2009).

*Mixonomatides.* Monophyly of Epilohmannioidea was only supported by the SH-aLRT test but is also supported by mitochondrial genome data (Arribas et al. 2020). Monophyly of Phthiracaroidea is also in accordance with earlier molecular studies (Domes et al. 2007a, b; Dabert et al. 2010; Arribas et al. 2020). Immatures of Epilohmannioidea and Phthiracaridae as well as those of Euphthiracaroidea dig tunnels in wood and plant litter and our results support that this is a synapomorphy of these superfamilies as suggested earlier (Norton and Behan-Pelletier 2009).

*Palaeosomatides.* Monophyly of Acaronychoidea is in accordance with earlier studies using 18S rDNA-based phylogenies (Maraun et al. 2009; Schaefer et al. 2010; Pachl et al. 2021). However, our analysis only included members of Acaronychidae but not Archeonothridae, and therefore monophyly of Acaronychoidea needs further investigation.

### Paraphyletic and polyphyletic non-brachypyline lineages

*Desmonomatides.* Our phylogeny indicated Crotonioidea to be paraphyletic since they included Collohmannioidea, and since Crotoniidae and Nanhermanniidae were the sister clade to Brachypylina. The placement of Collohmannioidea within Crotonioidea is consistent with earlier phylogenies based on 18S rDNA (Dabert et al. 2010; Pachl et al. 2021). Monophyly of Crotonioidea is controversial; they have been found to be paraphyletic based on 28S rDNA (Maraun et al. 2004), whereas they were monophyletic based on small and large subunits of the rDNA (Pepato and Klimov 2015) and mitochondrial genomes (Arribas et al. 2020).

*Enarthronotides.* Paraphyletic grouping of Atopochthonioidea and Heterochthonioidea is consistent with earlier morphology-based suggestions (Norton 2001; Norton and Behan-Pelletier 2009). The positions of atopochthoniid taxa were weakly supported and monophyly of Atopochthonioidea was inconsistent across molecular studies that used 18S rDNA (Schaefer et al. 2010; Schäffer et al. 2020; Pachl et al. 2021). Norton and Behan-Pelletier (2009) assigned *Pterochthonius* and *Atopochthonius* to separate families, i.e., Pterochthoniidae and Atopochthoniidae, suggesting that e.g., squat form and extended, foveolate setae evolved convergently. Heterochthonioidea are characterized by erectile setae similar to those of the protoplophorid lineage Cosmochthonioidea (Norton and Behan-Pelletier 2009). Since Heterochthonioidea include a paraphyletic family, i.e., Trichthoniidae (Norton and Behan-Pelletier 2009) and in our analysis Heterochthonioidea were paraphyletic and only distantly related, this character may have evolved convergently.

*Mixonomatides*. In our study, Epilohmannioidea were the sister group of Euphthiracaroidea and Phthiracaroidea (Pepato and Klimov 2015). Members of the families Euphthiracaridae and Synichotritiidae are characterized by a holoventral plate, i.e., the fusion of the four ventral plates (Norton and Behan-Pelletier 2009). According to Norton and Behan-Pelletier (2009), Synichotritiidae evolved the holoventral plate independently from Euphthiracaridae. However, since they were sister taxa in our study, this trait may have only evolved once (but see Norton and Lions 1992).

*Palaeosomatides.* In our analysis Ctenacaroidea and Palaeacaroidea were paraphyletic but closely related supporting their fusion into one superfamily i.e., Palaeacaroidea, as proposed by Subías (2004, 2022).

*Parhyposomatides.* As indicated by our study, Parhypochthonioidea are diphyletic which is in accordance with 28S rDNA data (Maraun et al. 2004). In the latter study, some taxa of Parhypochthonioidea grouped together with the mixonomatid lineage Nehypochthonioidea, a relationship also proposed by Woas (2002), who assigned the family Nehypochthoniidae to Parhypochthonioidea. However, in our study Nehypochthonioidea were not closely related to Parhypochthonioidea but formed the sister lineage to the more derived Desmonomatides, arguing against Woas’ (2002) suggestion.

### Monophyletic superfamilies in Brachypylina

As indicated by our study, Achipterioidea are monophyletic if recent suggestions on transferring *Lepidozetes* to Ceratozetoidea are accepted (Seniczak et al. 2014; Subías 2004, 2022). Since morphological (Seniczak et al. 2014) and molecular data (Maraun et al. 2009) agreed on placing *Lepidozetes* into Ceratozetoidea, we consider Achipterioidea a well-supported monophylum, which is in accordance with earlier molecular data (Maraun et al. 2004; Schäffer et al. 2010, 2020).

Monophyly of Carabodoidea is in accordance with earlier phylogenies based on 18S rDNA, 28S rDNA and other genes (Maraun et al. 2004; Dabert et al. 2010; Schäffer et al. 2010). Members of the carabodoid genera *Dolicheremaeus* and *Beckiella* share specific respiratory taenidia (Norton and Behan-Pelletier 2009) with aquatic taxa of Limnozetidae and Ameronothridae (Travé 1986; Norton and Behan-Pelletier 2009), suggesting that these adaptations evolved convergently at least 3 times.

Galumnoidea including the genera *Acrogalumna*, *Galumna* and *Pergalumna* were monophyletic, which is in accordance with mitochondrial genome data (Arribas et al. 2020) and 18S-based phylogeny (Schäffer et al. 2020). By contrast, Galumnoidea were found to be paraphyletic when *Allogalumna* was included (Dabert et al. 2010). Therefore, more taxa of Galumnoidea need to be included to resolve the monophyly of Galumnoidea.

As indicated by our study, Hermannielloidea were monophyletic. Combining Hermanniellidae and Plasmobatidae into one lineage was controversial (Grandjean 1962; Norton and Behan-Pelletier 2009) but is supported by our study and earlier phylogenies based on 18S rDNA and mitochondrial genomes (Arribas et al. 2020; Schäffer et al. 2020). Adults of both families carry exuviae and this may be a synapomorphic character (Norton and Behan-Pelletier 2009). By contrast, immatures of Hermanniellidae dig tunnels into dead wood (Luxton 1972), similar to immatures of other oribatid mite lineages, e.g., the mixonomatid Epilohmannioidea, Euphthiracaroidea and Phthiracaroidea (Norton and Behan-Pelletier 2009; Ermilov 2011) as well as the brachypyline lineage Carabodoidea (Norton and Behan-Pelletier 2009), suggesting that this trait evolved convergently at least 3 times.

Limnozetoidea were indicated to be monophyletic and included species of Hydrozetidae and Limnozetidae. Common ancestry of these two families is controversial and Subías (2004, 2022) assigned Limnozetidae to Ceratozetoidea. Although monophyly of Limnozetoidea was weakly supported in our study, earlier 18S-based phylogenies also supported monophyly of this lineage (Schäffer et al. 2020; Pachl et al. 2021).

Neoliodoidea were monophyletic and formed the basal lineage of Brachypylina. This is in accordance with other molecular studies (Maraun et al. 2004; Schäffer et al. 2010), and supports the view of Neoliodoidea being a basal taxon of Brachypylina as proposed earlier based on the structure of their respiratory organs (Norton and Alberti 1997; Norton et al. 1997; Norton and Behan-Pelletier 2009). Species of both lineages have macropores and minute sacculi on the notogaster (Norton et al. 1997; Norton and Alberti 1997; Norton and Behan-Pelletier 2009).

Oribatelloidea were indicated to be monophyletic and this is in accordance with earlier studies based on 28S rDNA (Maraun et al. 2004). This supports the exclusion of *Paralamellobates* from Oribatelloidea (Norton and Behan-Pelletier 2009; Behan-Pelletier et al. 2016).

Oripodoidea were monophyletic which is conform to other molecular phylogenies based on 18S rDNA, 28S rDNA and mitochondrial genomes (Maraun et al. 2004; Arribas et al. 2020; Li and Xue 2019; Pachl et al. 2021), although the assumed autapomorphy, i.e. the excentrosclerotic immatures, is not known for all members (Norton and Behan-Pelletier 2009). Several families within Oripodoidea were paraphyletic in our study, e.g., Haplozetidae and Scheloribatidae, and the position of *Protoribates* could not be resolved (see Fig. S1).

Monophyly of Phenopelopoidea as indicated by our study is in accordance with earlier molecular studies (Schäffer et al. 2010, 2020; Arribas et al. 2020). Norton and Behan-Pelletier (2009) included the family Unduloribatidae into Phenopelopoidea. By contrast, Subías (2004, 2022) assigned Unduloribatidae to Unduloribatoidea. Phenopelopoidea were paraphyletic when a taxon of Unduloribatidae was included (Schäffer et al. 2010). Therefore, the monophyly of Phenopelopoidea remains open since Undoloribatidae were not included in our study.

Tectocepheoidea were monophyletic which is in accordance with earlier molecular studies (Schäffer et al. 2010, 2020; Pachl et al. 2021). This argues against the inclusion of Tectocepheoidea within Carabodoidea (e.g., Balogh and Balogh 1992), as already suggested based on differences in juvenile morphology (Norton and Behan-Pelletier 2009).

### Paraphyletic and polyphyletic superfamilies in Brachypylina

In our analysis, Ameroidea were polyphyletic because a Caleremaeidae clustered within Damaeoidea, and a Rhynchoribatidae clustered within Oppioidea, also resulting in Damaeoidea and Oppioidea being paraphyletic. The inclusions of Rhynchoribatidae in Oppioidea is consistent with earlier suggestions based on morphological (Norton and Behan-Pelletier 2009) and genetic evidence (Schäffer et al. 2020).

In our study, Ameronothroidea were polyphyletic, since Ameronothridae were not associated with the well-supported clade comprising Selenoribatidae and Fortuyniidae (Norton and Franklin 2018; Pfingstl et al. 2023). Therefore, Selenoribatidae and Fortuyniidae, which are associated with littoral marine habitats in tropical regions, were separated from *Ameronothrus* spp., which are also occurring in such habitats (but are also terrestrial), and also from *Halozetes*, which are marine and terrestrial, and from the genus *Aquanothrus*, which occurs in freshwater (Marshall and Convey 2004).

Cepheusoidea were paraphyletic which is consistent with earlier studies (Maraun et al. 2004; Schäffer et al. 2010). Eutegaeidae had been transferred to Cepheusoidea (from Polypterozetoidea) based on immature resemblance (Marshall et al. 1987; Norton and Behan-Pelletier 2009; Schatz et al. 2011), but it had also been placed as separate superfamily Eutegaeoidea (Luxton 1988; Balogh and Balogh 1992; Colloff 2023). Based on our results, it is neither closely related to Cepheusoidea nor to Polypterozetoidea. Further, the families Microtegaeidae and Anderemaeidae (both Cepheusoidea) were basal to Carabodoidea in our analysis, together forming a well-supported clade. Microtegaeidae were proposed to be closely related to the cepheoid family Eutegaeidae (Woas 2002) but also to be distinct from cepheoids (Colloff 2019). Further, Microtegaeidae were included in Charassobatoidea (Subías 2004, 2022), a superfamily not recognized by Norton and Behan-Pelletier (2009).

Ceratozetoidea were polyphyletic, however, the families Ceratozetidae, Chamobatidae, Humerobatidae and Punctoribatidae formed a well-supported clade together with *Halozetes* and the monophyletic lineages Oribatelloidea, Phenopelopoidea and Galumnoidea. Close association of these lineages has been found before (Maraun et al. 2004; Schäffer et al. 2010; Arribas et al. 2020), indicating that Ceratozetoidea are basal to Galumnoidea, Oribatelloidea and Phenopelopoidea. Only *Paralamellobates* was not part of the Ceratozetoidea clade in our analysis but was closely related to Ameronothroidea.

Eremaeoidea were polyphyletic, which is in accordance with an earlier molecular study (Schäffer et al. 2010). In some classifications, Zetorchestidae were isolated as Zetorchestoidea (Marshall et al. 1987; Balogh and Balogh 1992; Subías 2004, 2022). However, in our study *Zetorchestes* was associated with *Ceratoppia* (Gustavioidea), although only supported by the SH-aLRT analysis. *Zetorchestes* and some peloppiid taxa (Gustavioidea), e.g., *Ceratoppia,* are known to have the ability to jump (Norton and Behan-Pelletier 2009). *Zetorchestes* has modified legs IV, i.e., jumping legs (Norton and Behan-Pelletier 2009), a character that has also been described for the peloppiid genus *Ceratorchestes* (Balogh and Mahunka 1969) a close relative of *Ceratoppia*. Ermilov and Kalúz (2012) questioned the modification of legs IV as a generic character for the peloppiid taxon. However, according to our analysis the ability to jump may be a synapomorphy of Zetorchestidae and Peloppiidae. Arceremaeidae had been included in Eremaeoidea based on the similarity of immatures (Woas 2002; Norton and Behan-Pelletier 2009), but in our study they were separated from Eremaeoidea.

Gustavioidea were polyphyletic; one clade comprised the families Liacaridae, Tenuialidae and Xenillidae, while the families Ceratoppiidae (*Ceratoppia bipilis*) and Astegistidae (*Cultroribula bicultrata*) were separated from the other Gustavioidea. Paraphyly of this superfamily is in accordance with earlier molecular studies (Maraun et al. 2004; Dabert et al. 2010; Schäffer et al. 2010) in which Ceratoppiidae were not associated with Liacaridae. Morphological studies also stressed that immatures and adults of these families are distinct (Seniczak and Seniczak 2010).

Licneremaeoidea were polyphyletic which is in accordance with molecular studies that included the licneremaeoid families Scutoverticidae and Charassobatidae (Schäffer et al. 2010, 2020). In our study, the association of Licneremaeidae and Charassobatidae indicates a close relationship between these families as hypothesized based on morphological data (Grandjean 1958; Norton and Behan-Pelletier 2009).

Polyphyly of Oppioidea was due to separate clustering of Thyrisomidae which is in accordance with earlier molecular studies (Maraun et al. 2004; Schäffer et al. 2020; Pachl et al. 2021). A possible relationship between Thyrisomidae and the lineage Gustavioidea as suggested by Woas (2002) could not be confirmed in our study.

Plateremaeoidea were diphyletic because taxa of the family Gymnodamaeidae were separated from a licnodamaeid taxon similar to the phylogeny of Schäffer et al. (2020). Subdivision of Plateremaeoidea into two lineages has been proposed earlier, with the first being Gymnodameoidea including Gymnodamaeidae, and the second being Plateremaeoidea including, among others, Licnodamaeidae (Bayartogtokh and Smelyansky 2004; Subías 2004, 2022). In our study, Licnodamaeidae were associated with *Cymbaeremaeus cymba* (Cymbaeremaeoidea) with high support and close relationship between these two lineages has been suggested earlier (Grandjean 1931; Ermilov and Stary 2021). As *Cymbaeremaeus cymba* (Cymbaeremaeoidea) was not associated with the two *Scapheremaeus* (Cymbaeremaeoidea) taxa included in our study, Cymbaeremaeoidea are likely to be paraphyletic as suggested earlier (Maraun et al. 2009; Schäffer et al. 2010, 2020).

### Endeostigmata

As indicated by our analysis, four of the five endeostigmatid superfamilies are likely to be monophyletic (Alycoidea, Alicorhagioidea, Oehserchestoidea and Terpnacaroidea), but support for the monophyly of Alycoidea was less strong. Nematalycoidea were polyphyletic.

Monophyly of Alycoidea is in accordance with Pepato and Klimov (2015) and Pachl et al. (2021) but contrasts the phylogeny of Dabert et al. (2010). Monophyly of Oehserchestoidea and Terpnacaroidea supports the establishment of these taxa by Grandjean (1939) and Kethley (1977), respectively. Monophyly of Alicorhagioidea agrees with the view of Grandjean (1939). Alicorhagioidea, Terpnacaroidea and Oehserchestoidea were only distantly related in our study indicating that particulate feeding of immature stages likely evolved several times convergently. Polyphyly of Nematalycoidea was due to *Micropsammus* (Nematalycoidea) forming a separate clade which is in accordance with earlier studies (Pepato and Klimov 2015; Pachl et al. 2021; Pepato et al. 2022).

In our study, the endeostigmatid lineages Oehserchestoidea and Terpnacaroidea, and the taxa *Stigmalychus* (Alicorhagioidea) and *Micropsammus* (Nematalycoidea) were part of Oribatida. This contrasts with Pachl et al. (2021) where Alycoidea, *Alicorhagia* (Alicorhagioidea) and *Micropsammus* (Nematalycoidea) were part of Oribatida. Overall, existing molecular phylogenies support the paraphyly of Endeostigmata, but the relationship between endeostigmatid and early-derived oribatid mite lineages need further attention. Astigmata clustered in oribatid mites (as siter taxon to Hermanniidae) and were monophyletic as has been suggested earlier (Maraun et al. 2004).

## Conclusions

Overall, our findings support monophyly of 17 of the 41 oribatid mite superfamilies recognized by Norton and Behan-Pelletier (2009), but also indicate that 18 superfamilies are not monophyletic. Furthermore, monophyly of four of the five Endeostigmata lineages and that of Astigmata is supported. The frequent polyphyletic groupings infer that convergent evolution of traits has been important in Oribatida and Endeostigmata. Convergent evolution is ubiquitous in the tree of life. It generally points to the importance of ecological constraints of organisms facing similar ecological challenges (Conway Morris 2003; McGhee 2011). Similar constraints and convergent evolution may lead to a mosaic-like distribution of traits across the phylogenetic tree, especially in old taxa such as oribatid mites (Woas 1998). Future studies using a set of conserved phylogenomic marker genes or complete genomes may further resolve the relationships among oribatid mite superfamilies as well as the relationship between Endeostigmata, Astigmata and Oribatida.

## Supplementary Information

Below is the link to the electronic supplementary material.

Supplementary file1 (PDF 97 KB)

Supplementary file2 (PDF 85 KB)

Supplementary file3 (PDF 2020 KB)

Supplementary file4 (PDF 2030 KB)

Supplementary file5 (XLSX 30 KB)

## Data Availability

We declare all data is being provided within the manuscript.
