# Supplementary material for: Convergent evolution revealed by paraphyly and polyphyly of many taxa of oribatid mites: A molecular approach
Source: Exp Appl Acarol. 2024 Sep 8;93(4):787–802. doi: 10.1007/s10493-024-00960-1 (PMC11534895; doi:10.1007/s10493-024-00960-1)
Supplement: Supplementary file 1 — Supplementary file1 (PDF 97 KB) [file 10493_2024_960_MOESM1_ESM.pdf]

Figure S1: Hypothetical tree used for the constrained tree search illustrating possible phylogenetic relationships among the 41 oribatid mite superfamilies recognized by Norton and Behan-Pelletier (2009). Oribatida are highlighted in blue, Astigmata in red, Endeostigmata in green and the outgroup in black.

Figure S2: Maximum likelihood tree reconstructed with IQ-Tree v2.3.2 (Nguyen et al. 2015); for the collapsed version of the tree see Fig. 1. White circles indicate <95% ufBS / < 80% SH-aLRT support, black circles indicate ≥ 95 % ufBS / ≥ 80 % SH-aLRT support. Oribatida are highlighted in blue, Astigmata in red, Endeostigmata in green and the outgroup in black.

Figure S3: Maximum likelihood tree reconstructed with IQ-Tree v2.3.2 (Nguyen et al. 2015) using the constrained tree search option ('-g'). White circles indicate <95% ufBS / < 80% SH-aLRT support, black circles indicate ≥ 95 % ufBS / ≥ 80 % SH-aLRT support. Oribatida are highlighted in blue, Astigmata in red, Endeostigmata in green and the outgroup in black.
