# Supplementary figures and images for: Convergent evolution revealed by paraphyly and polyphyly of many taxa of oribatid mites: A molecular approach
Source: Exp Appl Acarol. 2024 Sep 8;93(4):787–802. doi: 10.1007/s10493-024-00960-1 (PMC11534895; doi:10.1007/s10493-024-00960-1)

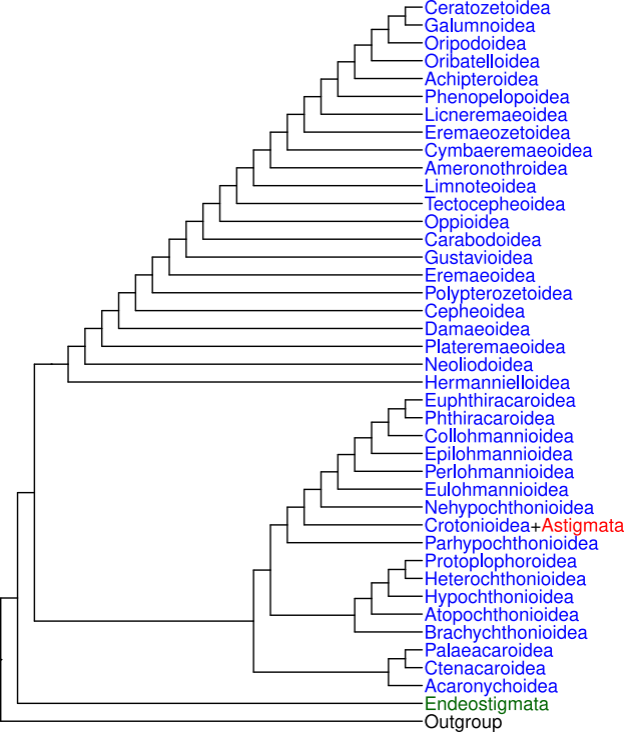

Supplement: Supplementary file 2 — Supplementary file2 (PDF 85 KB) [file 10493_2024_960_MOESM2_ESM.pdf]

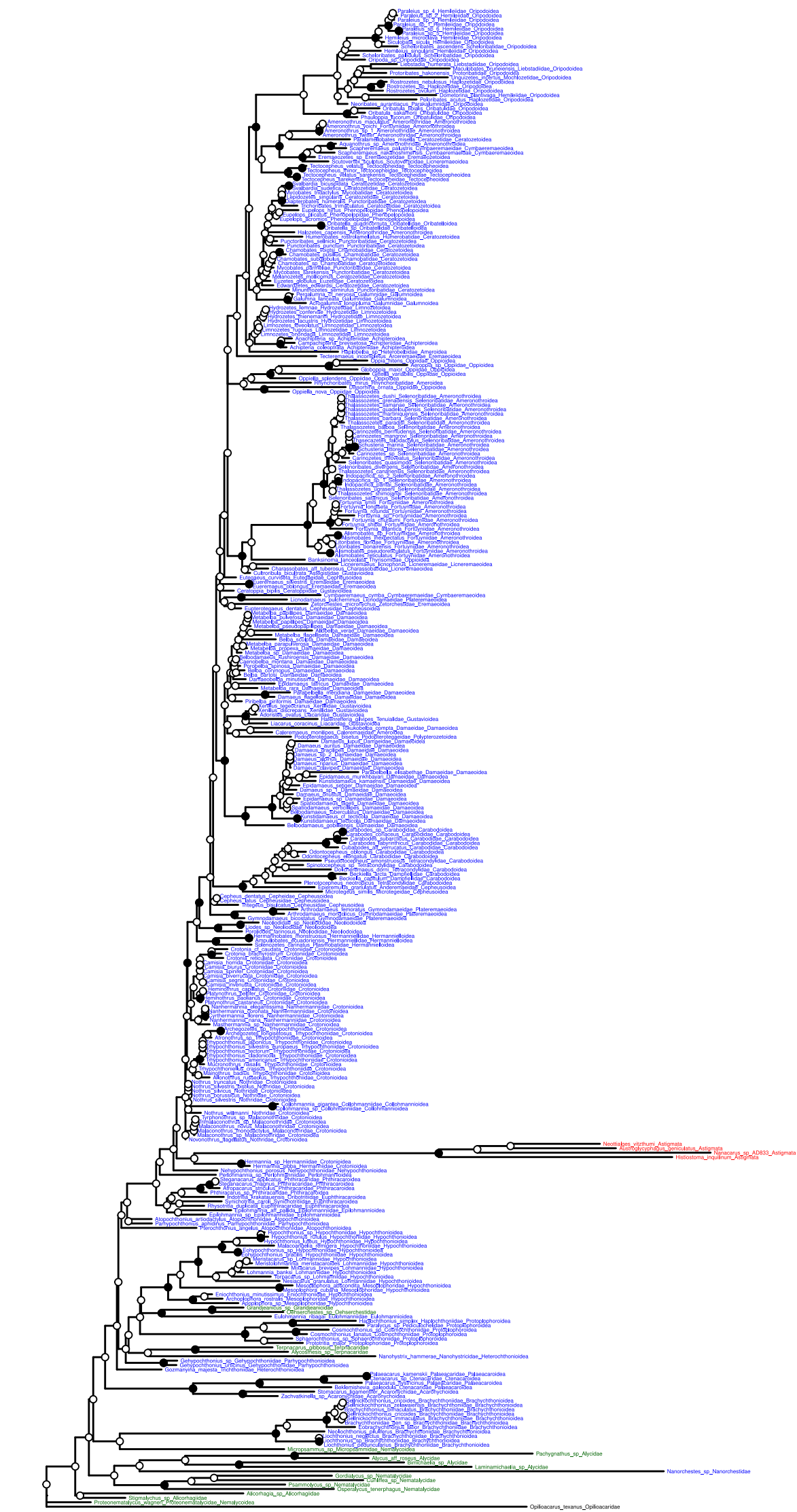

Supplement: Supplementary file 3 — Supplementary file3 (PDF 2020 KB) [file 10493_2024_960_MOESM3_ESM.pdf]

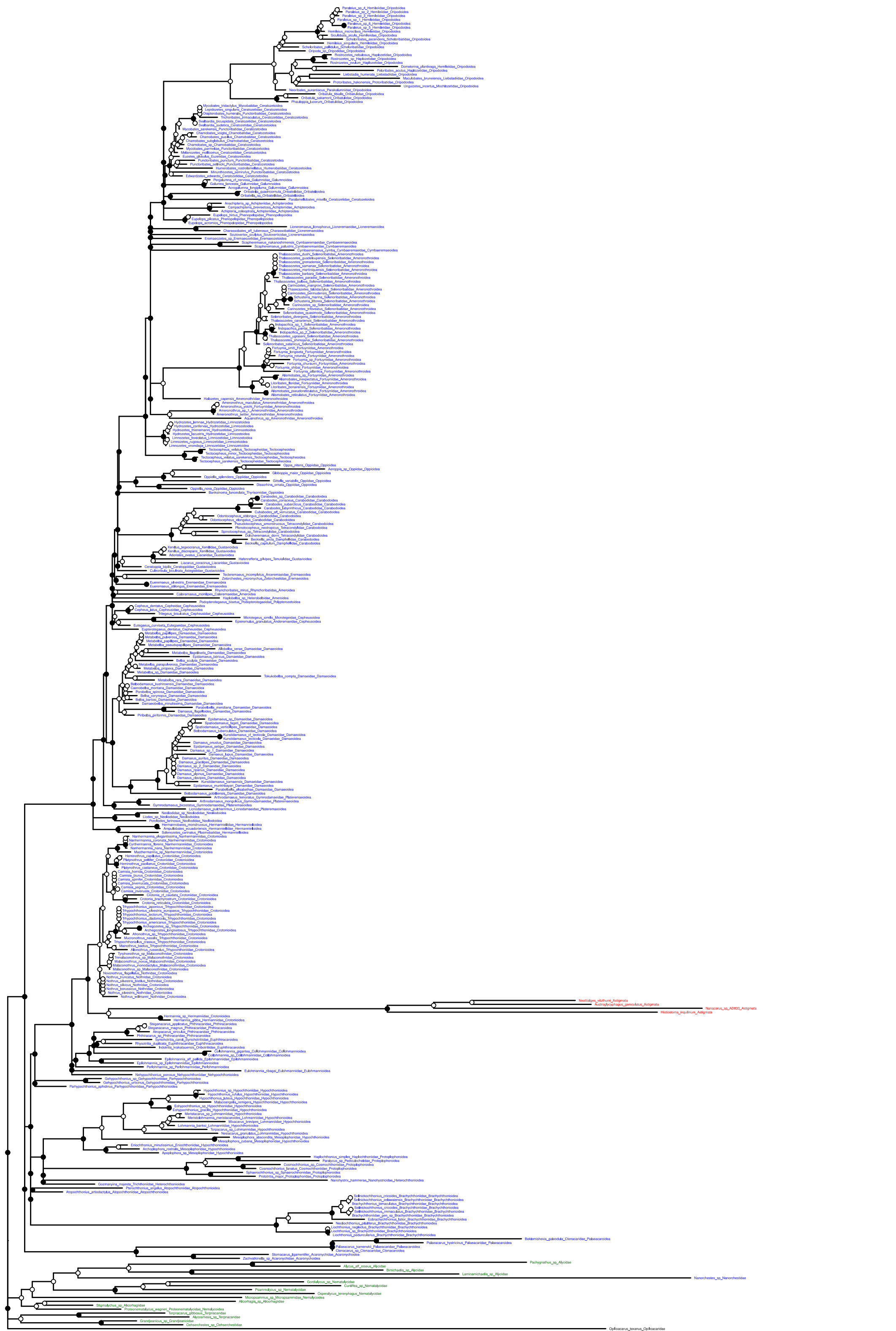

Supplement: Supplementary file 4 — Supplementary file4 (PDF 2030 KB) [file 10493_2024_960_MOESM4_ESM.pdf]
